# A mix-and-match reverse genetics system for evaluating genetic determinants of orthobunyavirus neurological disease

**DOI:** 10.1371/journal.pone.0315694

**Published:** 2025-04-28

**Authors:** Heini M. Miettinen, Matthew J. Abbott, Alyssa B. Evans

**Affiliations:** Department of Microbiology and Cell Biology, Montana State University, Bozeman, Montana, United States of America; George Mason University, UNITED STATES OF AMERICA

## Abstract

The encephalitic orthobunyaviruses have tri-segmented, negative sense RNA genomes and can cause severe neurological disease in humans, including La Crosse virus (LACV), which is the leading cause of pediatric arboviral encephalitis in the United States. However, little is known about the genetic factors that drive neuropathogenesis. Reverse genetics systems (RGS) are valuable tools for studying viral genetics and pathogenesis. Plasmid-based cDNA reverse genetics systems are available for LACV, however the plasmid backbones are medium-copy number and have a propensity for recombination. We therefore generated a plasmid-based cDNA reverse genetics system for LACV utilizing a more stable and high-copy number plasmid backbone. Additionally, we created the first full reverse genetics systems for two closely related orthobunyaviruses, Jamestown Canyon virus (JCV), and Inkoo virus (INKV), which have differing reported disease incidences in humans and differing neuropathogenic phenotypes in mice compared to LACV. We compared wild type (wt) viruses with RGS-derived wt viruses in human neuronal cells and in mice, and found that RGS-derived wt viruses maintained the replication and neuropathogenic phenotypes of their wt counterpart. Additionally, we demonstrated that reverse genetics plasmids from different parental viruses can be readily mixed-and-matched to generate reassortant viruses. This system provides a valuable genetic tool utilizing viruses with differing neuropathogenic phenotypes to investigate the genetic determinants of orthobunyavirus neuropathogenesis.

## Introduction

The Genus *Orthobunyavirus* contains many members capable of causing severe neurological disease in humans. These include closely related La Crosse virus (LACV), Jamestown Canyon virus (JCV), and Inkoo virus (INKV) in the California Serogroup (CSG; [1]). LACV is the leading cause of pediatric arboviral encephalitis in the USA and causes ~100 neuroinvasive cases per year. The mortality rate of LACV is ~1% and long-term sequelae including seizure disorders and cognitive defects are common in survivors. JCV is found in the USA and Canada and causes ~50 neuroinvasive cases per year, primarily in adults. INKV is primarily found in Scandinavia and has been reported to cause a handful of neuroinvasive cases [2]. Our previous studies on the neuropathogenesis of these viruses identified key differences in their ability to gain access to the brain from the periphery (neuroinvasion) and their ability to replicate and cause damage and disease in the brain (neurovirulence) [3,4]. We found that LACV was neuroinvasive with high neurovirulence, JCV was not neuroinvasive but with high neurovirulence, and interestingly, INKV was neuroinvasive but with low neurovirulence. The viral determinants that mediate neuropathogenesis are not well understood.

Orthobunyaviruses have three negative-sense, single-stranded RNA genome segments: L, M, and S. The L segment encodes the RNA-dependent RNA polymerase (L protein) [5]. The M segment encodes a polyprotein with the two envelope glycoproteins (Gn and Gc) and a nonstructural protein (NSm) of uncertain function [6,7]. The S segment encodes the nucleocapsid protein (N) which forms protective ribonucleoprotein complexes with the RNA genome, and nonstructural protein S (NSs), which is encoded within the N reading frame and results from leaky ribosomal scanning [7,8]. NSs is an interferon (IFN) antagonist [9] and the only known virulence factor for LACV.

Previous studies of orthobunyavirus genetic determinants of neuropathogenesis have largely relied on generating reassortant viruses using highly passaged attenuated viruses with unknown mutations [10,11]. More recent studies have utilized LACV reverse genetics systems to investigate the effect of targeted mutations, gene deletions, or ORF replacements in LACV neuropathogenesis [12–14]. While this approach provides critical insights into viral genetics, a limitation of this strategy to investigate viral determinants of neuropathogenesis is that mutations may disrupt protein functions in multiple ways. Viral proteins often serve multiple roles during infection, which can confound results and make teasing out mechanism from viral gene mutations and knockouts difficult.

To develop a reverse genetics system (RGS) that keeps viral proteins unchanged, we leveraged the key differences in neuropathogenic phenotypes between LACV, JCV, and INKV, to create full cDNA plasmid-based reverse genetics systems for each virus. We showed that RGS-derived wild type viruses maintained their replication and neuropathogenic phenotypes in human neuronal cells and mice compared to their wt counterpart. We also showed we can generate replication competent-derived reassortant viruses by mixing-and-matching L, M, and S RGS plasmids between the different viruses. With this system we generated a higher fidelity LACV reverse genetics system, as well as the first reported reverse genetics systems for JCV and INKV. This system provides a valuable genetic tool utilizing wild type versions of viral genes from viruses with disparate neuropathogenic phenotypes to investigate the viral determinants of orthobunyavirus neuropathogenesis.

## Materials and methods

### Cells and viruses

Gibco reagents were used unless otherwise specified. The BSR-T7/5 cells, a BHK cell line derivative that stably expresses T7 polymerase generated by Buchholz et al. [15], were maintained in GMEM supplemented 2mM L-Glutamine solution, 1x MEM Amino Acid Solution, and 10% FBS (Atlas Biologicals EF-0500-A), and every other passage with 1mg/ml Geneticin. Vero cells (ATCC) were maintained in DMEM supplemented with 10% FBS and 1x penicillin-streptomycin. SH-SY5Y cells (ATCC) were maintained in 1:1 ratio of EMEM (ATCC) and F-12K (ATCC) supplemented with 10% FBS and 1x pen-strep. LACV (human 1978), JCV (61V2235), and INKV (SW AR 83–161) were gifted by Dr. Karin Peterson (NIAID). Intermediate stocks of wild type (wt), RGS_wt, and RGS-derived reassortant viruses were generated in Vero cells with media supplemented with 25µg/ml of Plasmocin (Invivogen NC9886956) and harvested at 80% CPE at 2–4 dpi, then final stock viruses made in normal media.

### Generation of reverse genetics plasmids and viruses

Plasmids were constructed using our wt virus stocks and sequences of LACV human 1978, JCV 61V2235, and INKV SW-AR-83–161 [16]. All plasmids were generated in the pMK vector backbone. Full reverse genetics systems plasmids encoded full cDNA copies of the L, M, and S antigenome segments. Plasmids containing the full coding regions and UTRs of JCV and INKV M and S segments flanked by a T7 promoter and hepatitis delta virus ribozyme as previously described [12,13] were synthesized by Invitrogen GeneArt in the pMK vector backbone to generate reverse genetics plasmids pJCV-M, pJCV-S, pINKV-M, and pINKV-S. pMK plasmid vectors encoding the LACV L UTRs and portions of the INKV 3’ and 5’ coding regions or the JCV L UTRs and portions of the JCV 3’ and 5’ coding regions flanked by the T7 promoter and ribozyme were synthesized by GeneArt (pINKV-L_linker and pJCV-L_linker, respectively).

To make the LACV reverse genetics system plasmids, viral RNA from LACV-wt was isolated via Qiagen’s Viral RNA Mini Kit and cDNA generated using the iScript Select cDNA synthesis kit (Bio-Rad) and virus-specific primers (Eurofins; supplemental materials). Full-length cDNAs of LACV L, M, and S segments were made using Q5 High-Fidelity 2x Master Mix (New England Biolabs) using segment-specific primers with complementary 5’ overhangs with the vector backbone. The vector backbone was amplified using T7 promoter and ribozyme-specific primers. DNA assembly of the fragments was done using NEBuilder HiFi DNA Assembly Master Mix (New England Biolabs) at 50°C for 15 min. Two µl of the assembly mix was transformed into homemade chemically competent Stbl3 *E. coli*, which have a low rate of recombination due to mutations in the RecA gene, to generate pLACV-L, pLACV-M, and pLACV-S.

pINKV-L was generated using INKV-wt isolated RNA. cDNA was made using SuperScript IV VILO master mix (Invitrogen), and the coding region of INKV L amplified by PCR and purified using the Wizard SV Gel and PCR Clean-up System (Promega). The INKV L fragment and the pINKV-L_linker backbone were digested with *Aat* II and *Bsr* GI (New England Biolabs), purified, ligated with T4 DNA ligase (New England Biolabs) and transformed into homemade chemically competent Stbl3 cells. To generate pJCV-L, four fragments with 21–25 nucleotide overlaps encoding the JCV L segment were synthesized (GenScript). pJCV-L_linker was amplified using 2x Q5 high-fidelity master mix, digested with *Dp*n I to remove the template DNA and gel purified. DNA assembly was carried out using the NEBuilder HiFi DNA Assembly Master Mix for 60 min at 50°C. 2 µl assembly mix was transformed into 50 µl New England Biolabs 5-α chemically competent *E. coli* according to the manufacturer’s instructions. All plasmids were sequenced-verified by Plasmidsaurus and sequences analyzed with MacVector 18.5.1.

Transfections with the RGS plasmids were done by plating BSR-T7/5 cells at 1x10^5^ cells per well in a 6-well plate ~24 hours prior to transfection. Transfections were performed using TransIT-LT1 (Mirus MIR2304) according to manufacturer’s instructions with the following modifications: 0.5 µg of an L, M, and S plasmid and 9 µl TransIT-LT1 reagent was used per transfection mix. Supernatants were harvested and clarified at 48–72 hpt to recover RGS-derived wt viruses.

### Replication kinetics

Replication kinetics assays were performed as previously described [3]. Briefly, SH-SY5Y cells were plated at 1x10^5^ cells per well in 96-well plates. The next day, cells were inoculated in triplicate at MOI=0.01, incubated 1 hour at 37°C, washed twice, and media replenished. Supernatants were harvested and clarified at 1, 6, 12, 24, 48, 72, and 96 hpi, then titered on Vero cells. Three independent experiments were performed.

### Virus titers

Vero cells were plated in 24-well plates at ~1.3x10^5^ cells per well. The next day, virus was serially diluted 10-fold (replication kinetics) or 5-fold (virus stocks) and plated in duplicate in 200µl per well. Plates were incubated 1 hour at 37°C, then overlayed with 1.5% carboxymethyl cellulose (ThermoSci A18105.36) in MEM (Gibco 12360–038). Plates were fixed with 10% formaldehyde and stained with 0.35% crystal violet at 4 dpi.

### Mice

All mouse experiments were approved by Montana State University’s Institutional Animal Care and Use Committee under protocol number 2023–238-IA and were performed in accordance with NIH guidelines. Neuroinvasive disease was evaluated by inoculating weanling mice (21–23 days of age) intraperitoneally with 1x10^5^ PFU virus. Neurovirulence was evaluated by inoculating adult mice (>6 weeks of age) intranasally with 1x10^4^ PFU virus. Humane endpoint criteria of neurological disease, impaired movement that inhibited access to food and water, and moribundity was used. Signs of neurological disease included ataxia, seizures, limb paralysis or weakness, tremors, and circling. Non-neurological “sick rodent” signs of disease such as hunched posture and ruffed fur were not used as endpoint criteria. Mice were monitored at least twice daily during the potential clinical phase of the experiments. As soon as mice were observed displaying humane endpoint criteria, they were immediately humanely euthanized via overdose of isoflurane and exsanguination. Due to the neurological nature of disease, some animals have spontaneous seizures that result in death. In our experiments using 143 mice, 11 mice were found dead without displaying prior signs of disease. Of these, seven were adults inoculated intranasally (one with JCV-wt, one with INKV-RGS_wt, two with LACV-wt, and three with LACV-RGS_wt), and four were weanlings inoculated intraperitoneally (one with INKV-wt, one with LACV-wt, and two with LACV-RGS_wt). Of the remaining mice, 65 mice displayed humane endpoint criteria, and 67 mice never displayed humane endpoint criteria and were humanely euthanized by 25 dpi. Mice were group housed to reduce stress. Due to the nature of these experiments to evaluate pathogenicity, no analgesics or anesthetics were used during the study period as these could affect the study outcome.

### Statistical analyses

All statistical analyses were performed in GraphPad Prism v. 10.2. Replication kinetics were evaluated by two-way ANOVA with Sidak’s multiple comparisons test to determine differences between wt and RGS-derived wt viruses at any time point. Additional simple linear regression of slopes was performed for the log phase of viral growth at 6–72 hpi to compare 1) wt vs RGS-derived wt versions of the same virus and 2) all viruses compared to LACV-wt reference. Survival curves were analyzed via Mantel-Cox and Gehan-Breslow-Wilcoxon tests. For all analyses, p-values ≤0.05 were reported as significant.

## Results and discussion

### Generation of reverse genetics system-derived wt viruses

We utilized a strategy that has been used for other LACV reverse genetics systems (RGS) with individual DNA plasmids encoding cDNA copies of each of the L, M, and S antigenome segments under the control of a T7 promoter with a hepatitis delta virus ribozyme at the 3’ end [12,13]. Previous LACV RGS have utilized the pBR322 vector backbone. However, this medium-copy number plasmid has a propensity for recombination which can frequently result in plasmids with full or partial duplications, making targeted genetic changes and sufficient plasmid recovery a challenge. We therefore used a more stable high-copy number plasmid, pMK, as the backbone vector for our RGS plasmids. The pMK vector is from the ThermoFisher GeneArt plasmid expression vector pMX. It is derived from the pUC19 vector, which is a well-established high-copy number plasmid containing the pBR322 vector with mutations in the replication primer and deletion of the rop gene, which increases its copy number from the original pBR322 vector [17]. The full viral segments from LACV, JCV, and INKV L, M, and S segments were generated using our wild-type (wt) stock viruses (virus-wt) reference sequences. We used a variety of cloning and DNA synthesis techniques to generate plasmids pLACV-L, pLACV-M, pLACV-S, pJCV-L, pJCV-M, pJCV-S, pINKV-L, pINKV-M, and pINKV-S. All plasmids encode the full coding sequence and UTRs from each individual viral segment, with the exception of pINKV-L. This plasmid contains the full L coding sequence from INKV with the LACV L segment UTRs, due to insufficient sequence information on the INKV L segment UTRs. Plasmids were sequenced and compared to wt reference sequences. pLACV-L had a single nonsynonymous mutation, pJCV-L had two nonsynonymous and one synonymous mutation, pINKV-L had two nonsynonymous mutations, and pINKV-M had two nonsynonymous and one synonymous mutation (Table 1).

**Table 1:** Sequence comparison between the wt virus and the RGS plasmids.

Segment	pos	LACV-wt	LACV-RGS_wt	pos	LACV-wt	LACV-RGS_wt
L	2357	C	T	766	L	L
	pos	JCV-wt	JCV-RGS_wt	pos	JCV-wt	JCV-RGS_wt
L	4975	G	T	1641	***D***	***Y***
	4978	C	T	1642	***P***	***S***
	5523	C	T	1823	F	F
	pos	INKV-wt	INKV-RGS_wt	pos	INKV-wt	INKV-RGS_wt
L	2486	G	A	829	***R***	***Q***
	2808	G	A	936	***M***	***I***
M	2340	G	A	763	***R***	***K***
	2794	G	A	914	Q	Q
	3545	C	T	1165	L	L

### Nonsynonymous change

**Fig 1:**
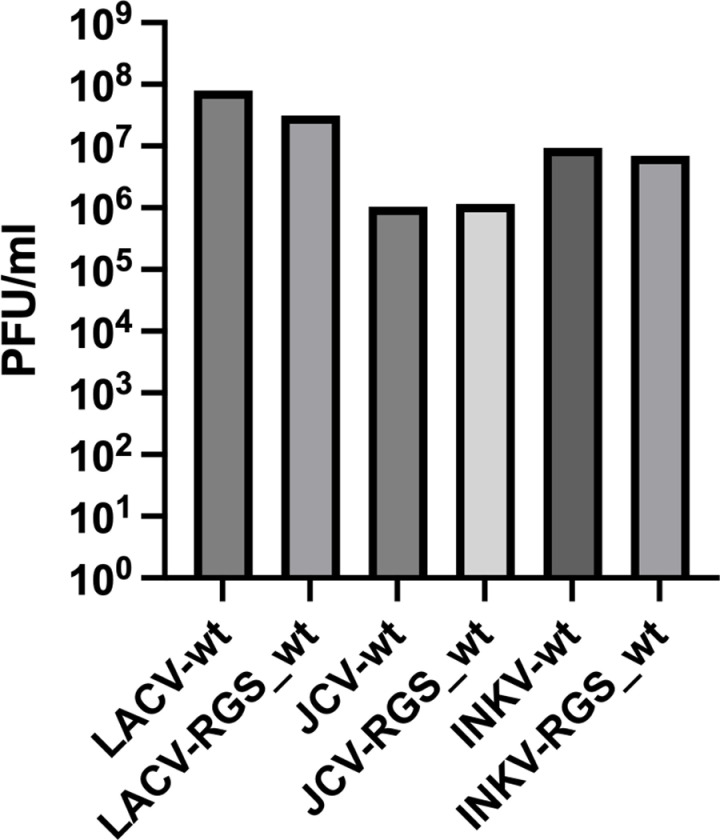
Comparison of wt and RGS-derived wt viral titers. Vero cells were infected with wt viruses and RGS_wt viruses at MOI=0.01. Viruses harvested at ≥80% CPE, then titered on Vero cells.

To generate RGS-derived infectious wt virus, the L, M, and S plasmids for each virus were transfected in the BSR-T7/5 cells that constitutively express T7 polymerase [15]. Supernatants containing the RGS-derived wt virus (“virus-RGS_wt”) were collected from the transfected BSR-T7/5 cells, and this supernatant was titered on Vero cells. To compare viral titers between wt and RGS-derived wt viruses, Vero cells were inoculated with each virus at MOI=0.01. At ≥80% CPE, the Vero-inoculated supernatants were harvested and subsequently titered on Vero cells. Both the wt and RGS_wt versions of each virus grew to similar titers (Fig 1; S1 File), suggesting that RGS_wt viruses maintain the replication and infectivity phenotypes of their wt virus counterpart.

### RGS-derived wt viruses maintain wild type replication phenotypes in neuronal cells *in vitro*

To further evaluate whether RGS-derived wt viruses replicated similarly to their wt counterpart, we performed replication kinetics assays. We have previously used SH-SY5Y neuroblastoma cells to compare the replication kinetics of wt LACV, JCV, and INKV and found that JCV and INKV replicated slower than LACV [3]. We therefore evaluated the replication kinetics of our wt and RGS_wt viruses in SH-SY5Y cells. We inoculated cells with each virus at an MOI=0.01 and collected supernatants at 1–96 hpi. Supernatants were titered on Vero cells.

**Fig 2:**
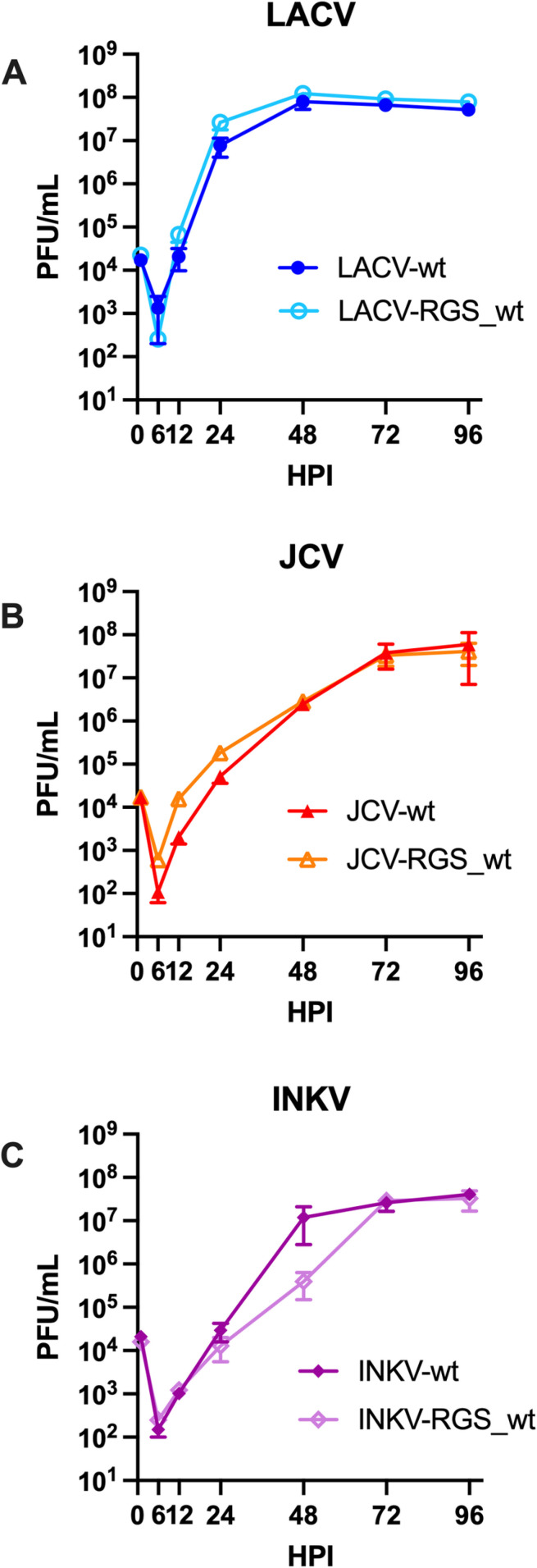
Replication kinetics of wt vs RGS_wt viruses in SH-SY5Y cells. Data represents three independent experiments titered on Vero cells.

Overall, the replication kinetics of wt vs RGS_wt viruses of the same virus were similar. There were some minor time point titer differences, however none of these were significantly different via two-way ANOVA (Fig 2; Table 2; S1 File). All wt and RGS_wt viruses of the same virus reached similar peak titers on the same day (Fig 2, Table 2; S1 File). Linear regression analysis found no significant differences in viral replication between wt or RGS_wt viruses of the same virus (Table 2). However, similar to our previous comparisons of wt LACV, JCV, and INKV, there were differences between virus strains in replication kinetics (Table 2). Both JCV and INKV viruses had significantly slower replication kinetics compared to LACV, consistent with our previous studies [3]. Together, these results indicate that the RGS-derived wt viruses maintain their wt replication phenotypes *in vitro*.

**Table 2:** Analysis of replication kinetics of wt and RGS_wt viruses.

	wt vs RGS_wt ANOVA p-values§	wt vs RGS_wt LR p-value☨	LACV-wt ref. LR p-value☨	Peak titer (PFU/ml)	Peak titer (hpi)
LACV-wt	0.63-0.98	0.282	ref	7.98 x 10^7^	48
LACV-RGS_wt			0.282	1.23 x 10^8^	48
JCV-wt	0.14-0.99	0.779	**0.032**	5.98 x 10^7^	96
JCV-RGS_wt			**0.013**	4.13 x 10^7^	96
INKV-wt	0.74-0.99	0.877	**0.007**	4.08 x 10^7^	96
INKV-RGS_wt			**0.007**	3.28 x 10^7^	96

§Two-way ANOVA with Sidak’s multiple comparison test.

☨Linear regression analysis of slopes covering the log phase of viral growth, 6–72 hpi.

### RGS-derived wt viruses maintain wild type neuropathogenic phenotypes in mice *in vivo*

**Fig 3:**
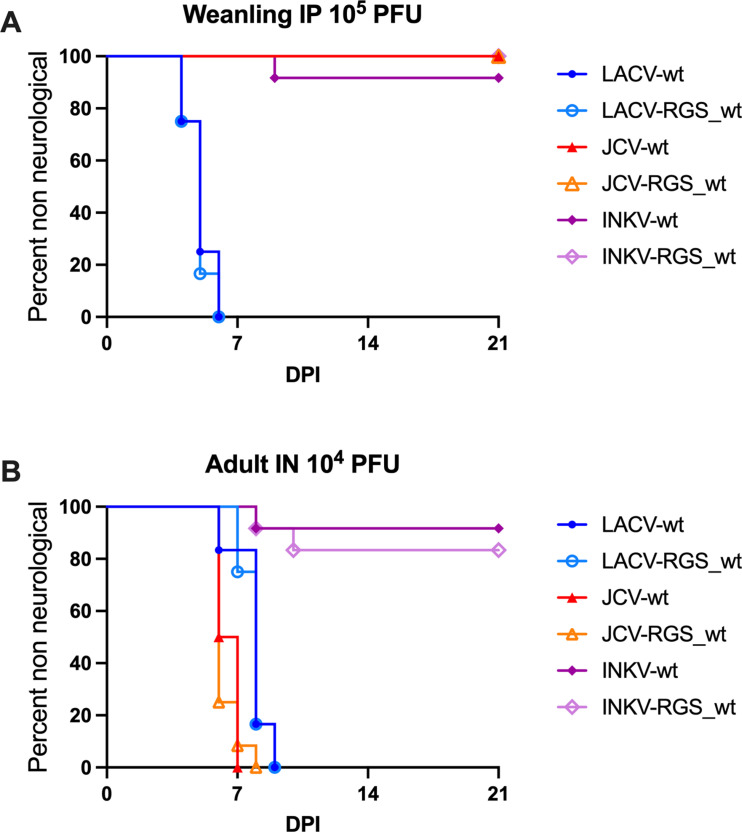
Comparison of wt and RGS_wt virus pathogenicity in mice. A) Weanling mice were inoculated intraperitoneally with 1x10^5^ PFU virus. B) Adult mice were inoculated intranasally with 1x10^4^ PFU virus. Mice were followed for neurological signs of disease. n=11-12.

We next evaluated if the RGS_wt viruses maintained their neuropathogenic phenotypes compared to their wt counterpart in mice. In our previous comparisons of LACV, JCV, and INKV of their ability to induce neuroinvasive disease after peripheral intraperitoneal (IP) inoculation in weanling mice. “Disease” refers to neurological signs or other endpoint criteria. We found that LACV caused disease in 100% of mice whereas JCV and INKV did not cause neuroinvasive disease in any mice [3]. We therefore inoculated weanling mice IP with 1x10^5^ PFU of the viruses to assess the development of neuroinvasive disease. Neurological signs included ataxia, paralysis, circling, and seizures. Both LACV-wt and LACV-RGS_wt induced neuroinvasive disease in 100% of mice at 4–6 dpi (Fig 3A). JCV-wt and JCV-RGS_wt did not cause neuroinvasive disease in any of the mice. INKV-RGS_wt did not cause neuroinvasive disease in any mice, however INKV-wt caused neuroinvasive disease in one out of twelve mice (Fig 3A). While we had not previously observed a mouse develop neuroinvasive disease from INKV, we have previously shown that INKV enters the brains of weanling mice in the absence of disease [3,4]. Therefore, it may not be surprising that occasionally an INKV-inoculated mouse may develop neuroinvasive disease. Despite this, there was no significant difference between any wt and RGS_wt virus of the same strain via simple survival curve analysis.

To evaluate neurovirulence phenotypes, we inoculated adult mice intranasally with 1x10^4^ PFU and followed them for neurological signs. At this route and dose, we have previously shown that LACV and JCV induced disease in 100% of mice, whereas INKV induced disease in ~15–20% of mice [3]. Consistent with previous results, LACV-wt caused neurological disease in 100% of mice at 6–9 dpi, and LACV-RGS_wt caused disease in 100% of mice at 7–9 dpi (Fig 3B). JCV-wt caused disease in 100% of mice at 6–7 dpi and JCV-RGS_wt caused neurological disease in 100% of mice at 6–8 dpi (Fig 3B). INKV-wt and INKV-RGS_wt induced neurological disease in one and two mice out of twelve, respectively (Fig 3B). There was no significant difference between any wt and RGS_wt virus of the same strain via simple survival curve comparison analysis.

Together, these results demonstrate that RGS-derived wt viruses maintain the neuropathogenic phenotypes of their wt counterpart, making RG-derived viruses an accurate tool for comparing differences in orthobunyavirus neuropathogenesis.

### RGS-derived reassortant viruses are successfully recovered from mix-and-match plasmid transfections

**Fig 4:**
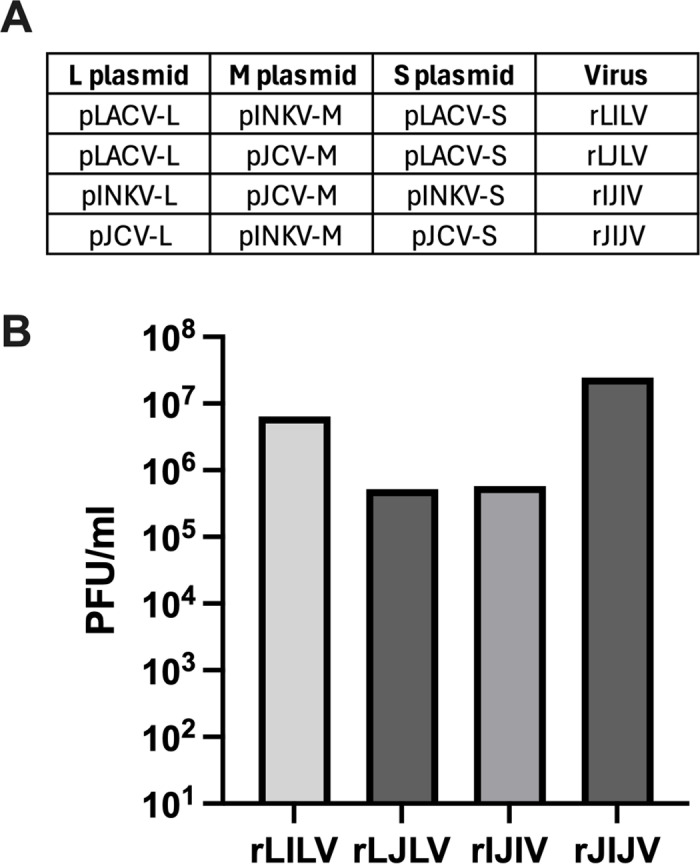
Generation of mix-and-match RGS-derived reassortant viruses. A) Table of the plasmids used for mix-and-match transfections in BSR-T7/5 cells to recover the reassortant viruses. B) Vero cells were infected at MOI=0.01 with wt, RGS_wt viruses, and RGS-derived reassortant viruses rLILV, rLJLV, rIJIV, and rJIJV. Viruses harvested at ≥80% CPE via visual inspection, then subsequently titered on Vero cells.

As proof-of-principal that we can generate reassortant viruses using this reverse genetics system, we next performed mix-and-match transfections between LACV & INKV, LACV & JCV, and JCV & INKV to generate M segment reassortant viruses rLILV, rLJLV, rIJIV, and rJIJV, which are summarized in Fig 4A. We titered the transfection supernatants, and then inoculated the RGS-derived reassortant viruses on Vero cells at MOI=0.01. The Vero cell supernatants were harvested at ≥80% CPE and subsequently titered on Vero cells in the same manner as previously described for the the wt and RGS_wt viruses (Fig 1). Consistent with the replication kinetics results, wt and RGS_wt viruses of the same strain had very similar titers (Fig 1). The RGS-derived reassortant viruses were more variable in titer than the parental viruses, but still reached titers of ~5x10^5^ – 2x10^7^ PFU/ml, depending on the virus (Figs 1, 4B). These results demonstrate that infectious virus can be recovered by mixing and matching L, M, and S plasmids from different parental viruses.

## Conclusions

Determining the underlying genetic factors that mediate orthobunyavirus neuropathogenesis is critical to understanding orthobunyavirus neurological disease and developing effective therapeutics and vaccines. To facilitate genetic comparisons between orthobunyaviruses with differing neuropathogenic phenotypes, we generated reverse genetics systems for LACV, JCV, and INKV. The three RGS-derived wt viruses maintained the neuropathogenic phenotypes of their wt counterparts both *in vitro* and *in vivo*. Furthermore, plasmids can be readily mixed-and-matched between parental viruses to generate reassortant viruses, making these an ideal tool for genetic studies of orthobunyavirus molecular pathogenesis.

## Supporting information

S1 File Data underlying the figures. Data for Figs 1 and 4 (Table 1): Titers (PFU/ml) of intermediate stocks grown in Vero cells inoculated with MOI=0.01 with either recovered transfection supernatants or wild type stock viruses. Titers determined by plaque assays in Vero cells. Data for Fig 2 and Table 2 (Table 2): Titers (PFU/ml) of time points for replication kinetics in SH-SY5Y cells. Supernatants were harvested and pooled from triplicate samples at each time point. The supernatants were titered via plaques assay in Vero cells. The titers for timepoints from three independent experiments for each virus are reported.
(XLSX)

## References

[1] Evans AB, Peterson KE. Throw out the map: Neuropathogenesis of the globally expanding California serogroup of orthobunyaviruses. Viruses. 2019;11(9):794. doi: 10.3390/v11090794 31470541 PMC6784171

[2] Putkuri N, Kantele A, Levanov L, Kivistö I, Brummer-Korvenkontio M, Vaheri A, et al. Acute human inkoo and chatanga virus infections, Finland. Emerg Infect Dis. 2016;22(5):810–7. doi: 10.3201/eid2205.151015 27088268 PMC4861510

[3] Evans AB, Winkler CW, Peterson KE. Differences in neuropathogenesis of encephalitic California serogroup viruses. Emerg Infect Dis. 2019;25(4):728–38. doi: 10.3201/eid2504.181016 30882310 PMC6433036

[4] Evans AB, Winkler CW, Peterson KE. Differences in neuroinvasion and protective innate immune pathways between encephalitic California serogroup orthobunyaviruses. PLoS Pathog. 2022;18(3):e1010384. doi: 10.1371/journal.ppat.1010384 35245345 PMC8926202

[5] Endres MJ, Jacoby DR, Janssen RS, Gonzalez-Scarano F, Nathanson N. The large viral RNA segment of California serogroup bunyaviruses encodes the large viral protein. J Gen Virol. 1989;70 ( Pt 1):223–8. doi: 10.1099/0022-1317-70-1-223 2732686

[6] Gentsch JR, Bishop DL. M viral RNA segment of bunyaviruses codes for two glycoproteins, G1 and G2. J Virol. 1979;30(3):767–70. doi: 10.1128/JVI.30.3.767-770.1979 480466 PMC353386

[7] Elliott RM. Orthobunyaviruses: Recent genetic and structural insights. Nat Rev Microbiol. 2014;12(10):673–85. 25198140 10.1038/nrmicro3332

[8] Fuller F, Bishop DH. Identification of virus-coded nonstructural polypeptides in bunyavirus-infected cells. J Virol. 1982;41(2):643–8. doi: 10.1128/JVI.41.2.643-648.1982 7077749 PMC256793

[9] Blakqori G, Delhaye S, Habjan M, Blair CD, Sánchez-Vargas I, Olson KE, et al. La Crosse bunyavirus nonstructural protein NSs serves to suppress the type I interferon system of mammalian hosts. J Virol. 2007;81(10):4991–9. doi: 10.1128/JVI.01933-06 17344298 PMC1900204

[10] Endres MJ, Griot C, Gonzalez-Scarano F, Nathanson N. Neuroattenuation of an avirulent bunyavirus variant maps to the L RNA segment. J Virol. 1991;65(10):5465–70. doi: 10.1128/JVI.65.10.5465-5470.1991 1895395 PMC249038

[11] Janssen RS, Nathanson N, Endres MJ, Gonzalez-Scarano F. Virulence of La Crosse virus is under polygenic control. J Virol. 1986;59(1):1–7. doi: 10.1128/JVI.59.1.1-7.1986 3712554 PMC253030

[12] Blakqori G, Weber F. Efficient cDNA-based rescue of La Crosse bunyaviruses expressing or lacking the nonstructural protein NSs. J Virol. 2005;79(16):10420–8. 16051834 10.1128/JVI.79.16.10420-10428.2005 PMC1182624

[13] Bennett RS, Gresko AK, Nelson JT, Murphy BR, Whitehead SS. A recombinant chimeric La Crosse virus expressing the surface glycoproteins of Jamestown Canyon virus is immunogenic and protective against challenge with either parental virus in mice or monkeys. J Virol. 2012;86(1):420–6. doi: 10.1128/JVI.02327-10 22013033 PMC3255902

[14] Hollidge BS, Salzano MV, Ibrahim JM, Fraser JW, Wagner V, Leitner NE, et al. Targeted mutations in the fusion peptide region of La crosse virus attenuate neuroinvasion and confer protection against encephalitis. Viruses. 2022;14(7):1464. doi: 10.3390/v14071464 35891445 PMC9317099

[15] Buchholz UJ, Finke S, Conzelmann KK. Generation of bovine respiratory syncytial virus (BRSV) from cDNA: BRSV NS2 is not essential for virus replication in tissue culture, and the human RSV leader region acts as a functional BRSV genome promoter. J Virol. 1999;73(1):251–9. 9847328 10.1128/jvi.73.1.251-259.1999 PMC103829

[16] Evans AB, Peterson KE. Cross reactivity of neutralizing antibodies to the encephalitic California serogroup orthobunyaviruses varies by virus and genetic relatedness. Sci Rep. 2021;11(1):16424. doi: 10.1038/s41598-021-95757-2 34385513 PMC8361150

[17] Lin-Chao S, Chen WT, Wong TT. High copy number of the pUC plasmid results from a Rom/Rop-suppressible point mutation in RNA II. Mol Microbiol. 1992;6(22):3385–93. doi: 10.1111/j.1365-2958.1992.tb02206.x 1283002

